# Audio-Visual Entrainment Neuromodulation: A Review of Technical and Functional Aspects †

**DOI:** 10.3390/brainsci15101070

**Published:** 2025-09-30

**Authors:** Masoud Rahmani, Leonor Josefina Romero Lauro, Alberto Pisoni

**Affiliations:** 1 School of Medicine and Surgery, University of Milano-Bicocca, 20126 Milan, Italy; 2 Department of Psychology, University of Milano-Bicocca, 20126 Milan, Italy; leonor.romero1@unimib.it (L.J.R.L.); alberto.pisoni@unimib.it (A.P.)

**Keywords:** audio-visual entrainment, neuromodulation, entrainment

## Abstract

Audiovisual Entrainment (AVE) is a non-invasive, non-pharmacological neuromodulation approach that aims to align brain activity with externally delivered auditory and visual rhythms. This review surveys AVE’s historical development, technical parameters (e.g., frequency, phase, waveform, color, intensity, presentation mode), components and delivery methods, reported clinical applications, and safety considerations. Given the heterogeneity of AVE protocols and terminology, we conducted a structured narrative review (PubMed, Scopus, Google Scholar; earliest records to July 2025), including human and animal studies that met an operational definition of regulated AVE and consistent administration of specified auditory and visual frequencies, with critical methodological details reported. We highlight AVE’s accessibility and versatility, outline a stepwise parameter reporting framework to support standardization, and discuss putative mechanisms via sensory and oscillatory pathways. However, current findings are heterogeneous and include null or limited effects. Mechanistic understanding and parameter optimization remain insufficiently developed, and premature claims of efficacy are not warranted. Rigorous, standardized, and adequately controlled studies are needed before AVE can be considered a reliable therapeutic tool.

## 1. Introduction

In the last two decades, neuromodulation techniques have drastically improved psychiatric disorder symptoms, either independently or in collaboration with other therapeutic methods. These techniques have captured the attention of scientists, leading to a dramatic increase in research publications in this field. However, several neuromodulation techniques remain underexplored, requiring further investigation to uncover their full potential.

One such technique is Audiovisual Entrainment (AVE). It has been studied for various disorders, with findings suggesting its potential therapeutic abilities. Despite this promise, challenges remain in understanding its mechanisms of action and optimizing protocol design due to the technique’s numerous components. Notably, AVE can engage most brain regions while being a versatile, cost-effective, and home-based solution that does not require clinical settings or specialized personnel. These characteristics underscore the importance of further studying AVE, refining its design, and understanding its effects to aid clinicians in therapeutic applications.

AVE is a non-pharmacological, rhythmic, stimulus-driven intervention that aligns the brain’s electrical frequencies with externally presented audio and visual cues [1]. This synchronization is thought to influence various cognitive and emotional processes. Furthermore, to improve conceptual understanding, distinctions have been established between audiovisual entrainment and audiovisual stimulation (AVS) to clarify the usage contexts of these terms: AVE involves a controlled, consistent delivery of targeted frequencies, while AVS may include exposure to a range of frequencies, such as those experienced during television watching [2].

Technically, AVE is delivered through devices ranging from essential headphones and visual displays to more sophisticated systems like AVE-specific wearable glasses. These technologies highlight AVE’s flexibility, simplicity, and portability, enabling its application in both traditional clinical environments and user-driven settings at home as it is findable in the market.

Unlike more noninvasive brain stimulation (NiBS) techniques, which require extensive setup and maintenance, AVE devices are standalone units that do not require ongoing consumable costs. This economic efficiency, combined with AVE’s broad applicational potential in therapeutic settings and cognitive research, positions it as a valuable tool in clinical and experimental neuroscience.

AVE could be categorized as a neuromodulatory technique. It may act through the entrainment of the neural population to the delivered audio/visual frequency, a process utilized across various scientific disciplines. Entrainment, originally described as a biological phenomenon where an organism exhibits a frequency-resonant response to a sequence of stimuli, has also been found in neural populations in response to external stimulation [3]. AVE is characterized by its multifaceted aspects and can be implemented in numerous forms. The term “AVE” concisely encapsulates the essence of this technique; however, its representation varies across different studies. Despite these variations, the field of neuromodulation faces a significant challenge due to the absence of standardized protocols and consistent terminology for AVE.

One theoretical framework that may help explain this phenomenon is Dynamic Attending Theory (DAT), developed by Mari Riess Jones and colleagues, elucidates how people direct their attention to rhythmic patterns in the environment. The central idea is that attention itself is rhythmic and can synchronize with predictable structures in speech, music, and movement. When events are highly regular, they support future-oriented attending, allowing listeners to anticipate when important moments will occur. By contrast, less regular events encourage analytic attending, where people focus on local details such as grouping or counting. A key concept in DAT is attunement—the alignment of internal attentional rhythms with external temporal patterns, which enables accurate perception, prediction, and coordinated action [4].

AVE has also been referred to in the literature as:

- Audiovisual Stimulation (AVS) [5,6,7,8,9,10,11,12].
- Brainwave Entrainment (BWE) [1,13,14,15].
- Mind Machine [16,17,18].
- Light and Sound Machines (LSM) [19,20].
- Light and Sound Stimulation [19,21,22].
- Audio Photic Stimulation (APS) [9,23,24,25].
- Brain Wave Synchronizer (BWS) [26,27].
- Haptic and Multimodal Rhythmic Stimuli [28,29].
- Multisensory Stimulation [30,31,32].

The significance of audio and visual stimulation has been deeply entrenched in history. The impact of visual stimulation may have started with the discovery of fire when our ancestors perceived the alteration of consciousness by staring at a flaming burning fire [19]. Knowledge about the effects of auditory stimulation and the effort to make instruments capable of producing beats has been dated to 5500–2350 BC [33].

In the Ancient Greek age, Apuleius (124–170 AD) fabricated a light stimulator with a pot. Ptolemy (100–170 AD) proclaimed that if you placed a spoke wheel by the sunlight and rotated it, the observer would see flickering lights and enter a euphoric state [34]. In the late 19th and early 20th centuries, Pierre Janet, a French psychologist, observed that spinning a spoke wheel reduced symptoms of depression, tension, and hysteria [35].

Auditory stimuli, particularly in the form of rhythmic pulses, play a significant role in various religious rituals, exemplified by the practices of Shamanism. This spiritual tradition, which focuses on establishing connections with the supernatural world, characteristically initiates its sacred ceremonies with drumming. The drumbeat precedes attaining a shamanic state of consciousness, a critical phase during which shamans should engage in healing, wellness, and communication with spiritual entities. The drum is perceived not merely as a musical instrument but as a transformative tool, metaphorically described as a horse that transports the shaman to other worlds [36].

In summary, compelling evidence suggests that ancient civilizations recognized the profound impact of visual and auditory stimuli on human consciousness. From the effects of gazing into a fire to the rhythmic drumming in shamanic rituals, these sensory experiences have shaped human interaction with the world. However, in contemporary society, the significance of such stimuli may need to be improved. This is attributed to the heightened threshold for sensory excitement, exacerbated by constant exposure to the intense and pervasive influences of modern multimedia devices. This evolution in sensory engagement reflects a significant shift in how humans perceive and respond to auditory and visual stimuli in the digital age.

This review aims to examine Audiovisual Entrainment (AVE), focusing on parameters, clinical applications, underlying mechanisms, and safety considerations. By identifying gaps in current knowledge and discussing potential future directions, it seeks to advance understanding and guide the standardization of AVE protocols for therapeutic and research purposes. Due to the diversity of AVE protocols, inconsistent terminology, and variability in delivery, a formal systematic review was not feasible. Instead, a structured narrative approach was employed. Relevant studies were identified through PubMed, Scopus, and Google Scholar searches, supplemented by manual reference checks. Search terms included audiovisual entrainment, audiovisual stimulation, and related terminology. Studies were included if they met our operational definition of AVE—regulated, consistent administration of specified auditory and visual frequencies, as outlined in the introduction and with critical methodological details reported. Both human and animal studies were considered, covering literature from the earliest available entries to July 2025.

## 2. AVE Parameters

The categorization of Audiovisual Entrainment (AVE) as a technique of the noninvasive brain stimulation (NiBS) techniques remains unclear. Nevertheless, AVE exhibits several standard parameters comparable to conventional NiBS methods, indicating potential parallels in their neuromodulatory impacts.

Various neuromodulation techniques, such as transcranial direct current stimulation (tDCS), employ multiple parameters influencing their functional mechanisms. In tDCS, aspects such as stimulation duration, the electric current’s polarity, electrode size, and current intensity are critical. This setting dramatically determines whether the stimulation produces inhibitory or excitatory effects on neural circuits [37]. Notwithstanding initial research, the comprehensive effects of various AVE configurations on brain responses and clinical outcomes remain ambiguous. Preliminary findings, like those by Rosenfeld [38] illustrate possible applications of specific AVE configurations while underscoring the necessity for additional research. Further comprehensive research is necessary to assess AVE’s therapeutic potential and to refine its parameters for certain clinical requirements, while the main components and parameters remain undefined and the approach is still far from a standardized technique.

Similarly, the effectiveness of AVE in influencing neural activity is contingent upon several specific parameters. These parameters include the visual pulse’s color and shape, the stimuli’s frequency and phase, and the auditory pulse’s pitch. Research exploring the modulation of psychiatric disorders by these parameters has shown that various settings can alter cortical activity effects to achieve precise therapeutic results [39,40,41].

Considering these similarities to conventional NiBS, it becomes essential to investigate if distinct attributes of AVE can independently affect brain activity. In the subsequent sections, the existing research on these elements will be examined, to assess their possible role in neuromodulation.

### 2.1. Intensity

The first essential element of the AVE that requires modification prior to initiation is the intensity of the light and the sound volume, personalized for each participant. Customization is crucial because of the large variations in retinal sensitivity, pupil size and the anatomy of eyelashes and eyelids among individuals, particularly those linked to ethnic origins [42]. Moreover, auditory sensitivity is distinctly individual [43], necessitating precise modulation of sound loudness to ensure an optimal and comfortable experience for every participant. The above factors are crucial, as failure to adapt them may result in diminished effectiveness, discomfort for the participant, and feelings of fatigue and aversion.

### 2.2. Color

**Figure 1:**
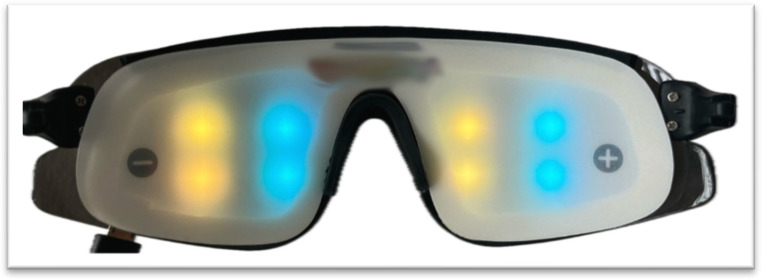
Example of color presentation through a commercial audiovisual entrainment (AVE) device. The device consists of eight light-emitting diodes (LEDs), with four LEDs positioned for each eye. For each eye, two LEDs target the left visual field and two LEDs target the right visual field. In this configuration, the left visual field is illuminated with yellow light, and the right visual field is illuminated with blue light. The colors shown are representative of a common experimental setup; yellow and blue were selected because different wavelengths can evoke distinct physiological and psychological responses, as reported in prior research [44,45,46].

Audiovisual Entrainment devices in the market typically incorporate lights of varying colors, as illustrated in Figure 1. A substantial body of research supports that color influences individual mood and behavior and elicits distinctly varied effects based on the specific hue [42,43,44]. For instance, studies have documented how different colors can induce different psychological responses, ranging from calming effects with blue to stimulating effects with red [45,46,47]. Moreover, the interaction between color and brain function extends beyond subjective psychological effects to quantifiable changes in brain activity. Münch [48] has shown that various colors can distinctly influence brain wave patterns. This evidence implies that the selection of colors in AVE devices could be deliberately tailored to engage specific neural rhythms linked with desired psychological states. These studies have important implications for the design and application of AVE devices. By understanding the nuanced effects of color on both psychological and neurophysiological levels, manufacturers can enhance the efficacy of AVE technologies. This suggests that, since different colors exert distinct psychological and neurophysiological effects, the choice of color in AVE studies should be made purposefully in line with the specific aims of the experiment. While we have highlighted some general differences in psychological responses (e.g., calming versus stimulating effects), researchers are encouraged to consult the existing literature in detail prior to study design to ensure that color selection is aligned with the intended outcomes.

### 2.3. Frequency

The frequency parameter is pivotal in noninvasive brain stimulation techniques, dramatically impacting neural outcomes. In the context of repetitive transcranial magnetic stimulation (rTMS), it has been documented that stimulation at a frequency of 1 Hz reduces cortical excitability, as demonstrated by Gerschlager [47]. Conversely, higher frequencies have an excitatory effect on cortical areas [48]. Similar variability in response based on frequency settings is observed in AVE. Research indicates that AVE stimulation within the theta frequency band (5 Hz) can enhance memory functions [49]. In contrast, stimulation in the beta frequency range appears to yield no significant improvements in cognitive performance [50]. This differential impact underscores the critical role of precise frequency selection in aligning with specific neurophysiological targets. Frequency settings directly influence the synchronization of brain networks by modulating brain oscillations and facilitating activity coordination across various neural nodes [51]. Therefore, the choice of frequency in AVE studies should be carefully considered and defined, drawing from an extensive review of the existing literature and the targeted therapeutic or cognitive outcomes. Such a strategic approach ensures that the selected frequencies are optimally aligned with the intended goals of the brain stimulation protocol.

### 2.4. Phase

**Figure 2:**
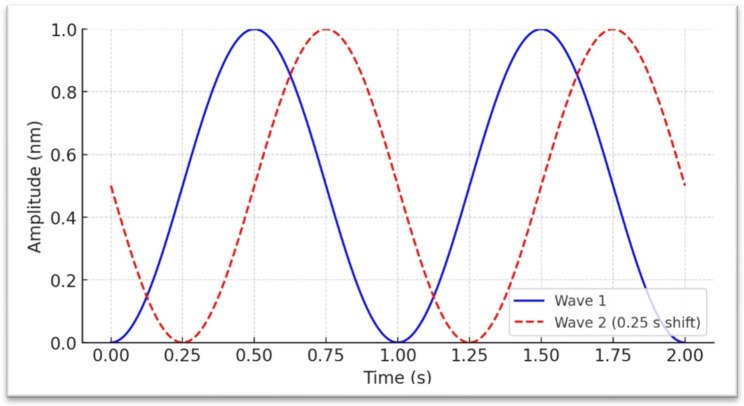
Depiction of two sinusoidal waveforms with amplitudes scaled from 0 to 1 nanometer (nm) and no interval between the flickers, allowing direct interpretation of displacement as a positive physical quantity. The blue solid waveform is phase-aligned to begin at (0 s, 0 nm), where it gradually rises to its peak in a smooth curve before descending back toward its minimum, forming a continuous oscillatory cycle characteristic of sinusoidal motion. The red dashed waveform is offset by 0.25 s relative to the blue waveform, illustrating a constant phase difference between the two signals. This phase arrangement could be applied to stimulate the right visual field of both eyes and the left visual field of the right eye, with the aim of selectively engaging the left and right brain hemispheres.

The visual processing system is unique in its contralateral organization. More specifically, stimuli from the right visual field are exclusively processed by the left hemisphere of the brain, while stimuli in the left visual field enter the right hemisphere [52]. Such an anatomical arrangement allows for selective, independent stimulation of each hemisphere. That is particularly valuable within neuromodulation techniques when one’s goal is to target some areas or functions of the brain. In the context of AVE, this neural architecture is exploited by stimulating the left visual field (LVF) and right visual field (RVF) with a phase offset, as illustrated in Figure 2. This method of stimulation results in differential brain responses, depending on the targeted hemisphere. Such hemisphere-specific stimulation can be particularly insightful for studies on understanding lateralized brain functions and their impact on cognitive and behavioral processes. Bunch of studies [53,54,55], supports the effectiveness of this approach, indicating that. Targeted visual field stimulation can elicit distinct patterns of brain activity, offering a nuanced tool for investigating and manipulating brain dynamics. This represents another important aspect of AVE, as it allows researchers to target more specific regions of the brain. However, such methodological details should always be explicitly reported in studies to ensure transparency and reproducibility.

### 2.5. The Shape of the Visual Pulse

Flicker refers to the rapid oscillation of light intensity, akin to the repetitive switching of a light source between on and off states. The duration of each flicker and the temporal dynamics required to achieve the lowest and highest intensity peaks can vary considerably. These variations generate diverse waveform patterns, which can differ in frequency, amplitude, and shape. These patterns influence the perceptual and physiological responses elicited by the flicker. This variation is attributed to how different waveforms interact with the neural dynamics of the brain. According to empirical finding [56] it has been demonstrated that sinusoidal waveforms (Figure 2), for instance, offer a smooth, continuous oscillation that might be less disruptive and more naturally integrated into the brain’s rhythms in contrast, square waveforms create a more abrupt, discrete transition between light and dark, which can induce a stronger neural response, particularly by enhancing cortical excitability and generating a more pronounced steady-state visual evoked potential. Triangular waveforms balance these two, with a linear but sharper change in light intensity compared to sinusoidal waves. It has been shown that the signal’s shape noticeably varies the entrainment’s effects by up to 50%. Beyond the primary effects of the waveform shape on entrainment, secondary parameters, often referred to as harmonic effects, also play a significant role. These effects are intricately linked to the fundamental waveform and arise from the overtones that each shape naturally produces. Harmonic effects can influence the complexity and the reach of entrainment across different brain regions, affecting both the efficacy and the stimulation experience [56]. Understanding the implications of these waveform characteristics is essential for optimizing flicker-based therapies and research protocols. It allows for designing more effective and tailored interventions to target specific neurological conditions or research objectives. For instance, choosing the appropriate waveform could enhance therapeutic outcomes and grant the safety in treatments for conditions like epilepsy, where precise control over neural rhythm modulation is crucial.

### 2.6. The Pitch of the Auditory Stimulus

Musical tones are primarily distinguished by pitch. This notion of pitch is essential for grasping auditory perception and has been the focus of scientific research for many years. Early research by Chatrian [57] explored how auditory clicks presented to one ear can differ significantly in perception and brain response, setting the stage for subsequent investigations into auditory processing. One seminal advancement in this field was introduced by oster [58] who investigated the phenomenon of auditory beat stimulation. This study showed that presenting two sinusoidal auditory waves at slightly different pitches to each ear led to distinct brain responses. This effect is known as binaural beats (BB), where each ear receives a different frequency, such as one ear receiving a tone at 200 Hz and the other at 210 Hz. The brain internally creates a perceptual third tone equal to the difference between the two frequencies. This internally generated beat is hypothesized to entrain brain activity, potentially increasing the amplitude of brain waves at the frequency of this beat. Subsequent research has explored how these auditory stimuli influence cognitive functions and are reflected in changes observed through electroencephalography (EEG). Engelbregt [59] examined both monaural beat (MB) and BB in 40 Hz. Findings showed that binaural beats improved attention while monaural beats impaired it. Despite these cognitive differences, EEG recordings showed no significant changes in gamma-band activity across conditions. Future research will likely continue to unravel these complex interactions, offering new avenues for theoretical insights and practical applications in neuropsychology and beyond. This component is another one which to what extant has been proven that able to change the effects and similar to the other parameters should be considered.

### 2.7. The Presentation Parameters

**Figure 3:**
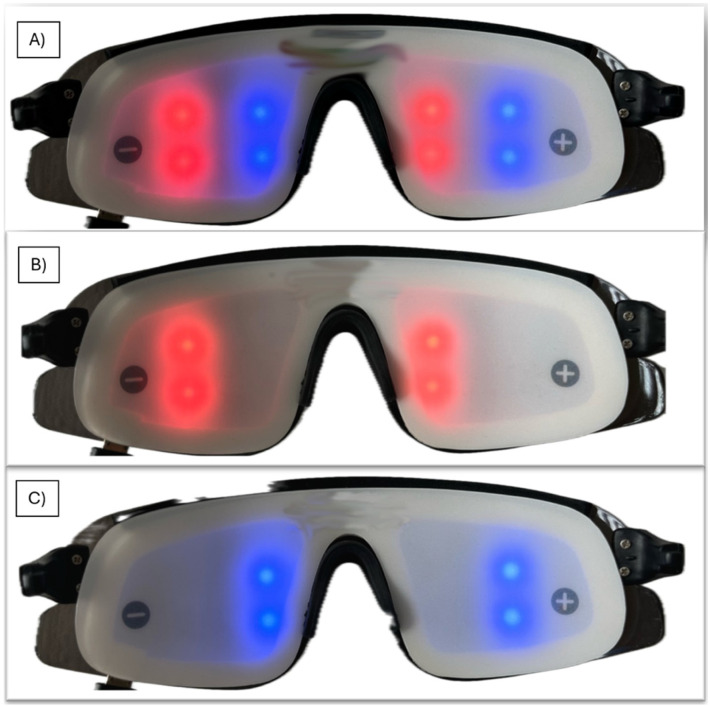
(**A**) delivering light into both eyes and visual fields. (**B**) Delivering into right visual field. (**C**) Delivering into left visual field.

The characteristics cited in the literature, including intensity, color, frequency, and phase, may affect the efficacy of AVE and potentially impact the results. Nevertheless, careful consideration must be afforded to the method in which the stimulation is delivered. One significant variable in the AVE application is the choice between eyes-open (EO) and eyes-closed (EC) conditions. Research indicates that these conditions activate distinctly different neural networks. A research by Han [60] illustrates these differences, showing that brain network connectivity significantly shifts between EO and EC conditions. With EO, AVE tends to increase connectivity in networks related to external sensory processing and attentional engagement. At the same time, EC conditions reduce external sensory integration, leading to enhanced connectivity in networks related to introspection and self-referential thought. These findings suggest that AVE can be tailored more effectively by adjusting visual engagement (EO or EC), depending on whether the goal is to stimulate outward-focused attention or inward-focused relaxation. Typically, AVE studies have utilized a conventional mode whereby visual stimuli are presented simultaneously to both eyes Figure 3A. This approach ensures uniform stimulus delivery across the visual fields, providing a baseline for observing generalized brain responses to visual entrainment.

In more specialized setups, stimuli can be directed separately to the right visual field (RVF) and left visual field (LVF), potentially even in different phases, as outlined in the phase section [61]. Another option is presenting two different frequencies which causes different dynamic phase. This method allows for the targeted stimulation of each brain hemisphere, as each visual field is primarily processed by the opposite cerebral hemisphere Figure 3B,C. This arrangement can investigate lateralized brain functions or address hemispheric imbalances in clinical scenarios. Further variations include delivering flickering light to only one eye or adjusting the frequencies for LVF/RVF stimulation. These nuanced approaches enable researchers to explore the specific effects of unilateral versus bilateral visual input on neural activity and cognitive functions.

In addition to visual stimuli, as mentioned auditory pulses in BB deliver auditory pulses at slightly different frequencies to each ear, producing a perceived beat frequency that arises from the differential in input. Conversely, monaural beats present a beat frequency directly. These auditory stimuli can be set at a fixed frequency, such as 10 Hz (as for transcranial alternative current stimulation (tACS)), or within a programmed range, like 13 to 18 Hz, mimicking parameters used in transcranial random noise stimulation (tRNS).

Alternatively, auditory stimuli can be administered randomly as introduced with white noise [62], which covers a broader frequency range. Depending on the frequency and randomness of the stimulation, such variability can induce different forms of brain plasticity or cognitive effects, potentially ramping up or down through different wavebands to elicit specific neural responses.

Each approach—visual or auditory, fixed or variable in frequency, eyes-open or eyes-closed—provides a unique toolset for manipulating neural activity through AVE. This versatility enhances the scope of research into brain function and Neurotherapy. It expands the potential clinical applications of AVE in treating neurological disorders, enhancing cognitive performance, or providing relaxation and stress relief. By tailoring the stimulation parameters, researchers and clinicians can optimize AVE methods to suit specific therapeutic goals or experimental needs, thereby advancing our understanding of the complex interactions between sensory stimuli and brain activity.

#### 2.7.1. Standardization Rationale and Step-Wise Reporting of Parameters

Up to now, the components and the presentation modes have been explained. In this section, instead, a step by step selection and consideration of the parameters is presented Table 1. Nevertheless, the current literature offers insufficient direct inquiry into each AVE parameter to determine the conditions under which it is most effective or appropriate Table 2. Disentangling these parameters and documenting the selection methods while justifying choices in light of contemporary knowledge would represent meaningful progress toward a standardized AVE protocol. This document provides a step-by-step guide for choosing and reporting the status and conditions of AVE components.

##### Step 1—Visual Stimuli

A critical aspect is the report of whether stimulation is delivered with eyes open or closed, and the specification of the spatial presentation within the visual fields. Flicker may be confined to one eye [63] or a single visual field, or may encompass both eyes and all four visual fields [61]. Specifying the stimulation frequency (or frequencies), including whether different frequencies are applied to different visual fields is crucial. As illustrated in Figure 2, flicker can be presented with controlled phase relationships; this is one method to differentially engage the cerebral hemispheres. The chosen frequency (or frequencies) may be static or variable over time [5]. For example, within the theta band (4–8 Hz), the stimulus may step from 4 Hz to 5, 6, 7, and 8 Hz in two-second epochs before returning to 4 Hz, or it may sample frequencies randomly from a predefined set. Color is an additional parameter that can modulate the effect and should be reported. Finally, the signal form (e.g., sinusoidal, square) is a critical element with meaningful consequences and must be specified [44,45,46].

##### Step 2—Auditory Presentation

Also auditory stimuli can be delivered to one ear or both. If bilateral, it must be specified whether tones/pulses are simultaneous or alternating between ears. It must also be indicated whether auditory beats are used (monaural or binaural) and report the pitch (carrier frequency) of the tones if applicable. Frequency is a key factor and should be documented with the same level of detail as for visual flicker (single vs. multi-frequency; static, stepped, swept, or randomized over time).

##### Step 3—Intensity Calibration (Link to Section 2.1)

A potential measure for standardization may involve evaluating each individual sensory threshold prior to presenting the AVE. Thresholds for both auditory and visual stimuli can be established by pinpointing the minimum level at which the stimulus is undetectable and the maximum level at which it is perceived as intrusive. The ideal configuration can subsequently be selected as the midpoint between these two values. The identical approach must be reiterated for the visual stimuli.

##### Step 4—Temporal Structure and Dose

The report have to include the total session duration, number of sessions, and weekly schedule. Indicate ramp-in/ramp-out periods to reduce startle and fatigue.

##### Step 5—Synchronization Across Modalities

The protocol must indicate whether auditory and visual streams are delivered at the same frequency, harmonically related, or intentionally offset; report any phase lead/lag (ms or degrees).

##### Step 6—Safety Checks and Exclusions

A pre-session screening (photosensitivity, migraine history, hearing issues), stop criteria, and any adverse events have to be included to control for potential adverse events or discomfort (see next section).

**Table 2:** This table presents AVE studies methodologically closest to the protocol proposed in this review. In all included studies, auditory and visual stimulation were delivered with defined timing and frequencies. Unreported parameters are indicated as not mentioned.

Study (Author Year)	Eyes Condition	Frequency (Hz)	Auditory Pulses (Pitch)	AVE Device Type	Phase (L/R Visual Fields)	Intensity (Audio/Visual)	Session Duration	Experiment Duration	Flicker Color
Adrian & Matthews (1934) [64]	Closed	10 Hz	Not mentioned	Photic stimulation goggles (early device)	Reported (details unclear)	Not mentioned	40 s	Not mentioned	Not mentioned
Brauchli et al. (1995) [5]	Closed	10 Hz	Not mentioned	Photic stimulation goggles (early device)	Reported (details unclear)	Not mentioned	40 s	Not mentioned	Not mentioned
Hsiung & Hsieh (2024) [6]	Closed	40 Hz	Not mentioned	LED light bulb and computer screen	Not mentioned	Not mentioned	3 min per group	10:00 a.m. to 9:00 p.m.	Not mentioned
Mansouri et al. (2022) [7]	Not specified	Not mentioned	Not mentioned	Repetitive light and sound exposure (cartoon sound, colored lights)	Not specified	Not specified	6 h/day	Behavioral tests	Not specified
Oppermann et al. (2023) [8]	Not specified	[7.8, 8.8, 12.8, 13.4, 14.4, 18, 19, 23] Hz	Not mentioned	Consumer-grade auditory-visual stimulation device	Not specified	Not specified	16 sessions over four weeks	Two lab sessions, rest at home	Not specified
Tang et al. (2014) [9]	Closed	Alpha to Delta (8 Hz to 1 Hz)	Not mentioned	Procyon AVS device (light goggles and headphones)	Not specified	Not specified	30 min nightly for 1 month	One-month intervention	Not specified
Làdavas, Tosatto, & Bertini (2022) [31]	Opened	Not mentioned	Not mentioned	Audio-visual stimulation with LEDs and loudspeakers	Not specified	Not specified	10 sessions (4 h/day)	2 weeks (Follow-up at 6.5 months)	Not specified
Locke et al. (2020) [15]	Opened	1 Hz to 23 Hz	Binaural beats, visual stimulation	Smartphone app with VR headset and headphones	Not specified	Not specified	10 min per day (up to 40 min max)	4 weeks at home	Not specified
Teplan et al. (2009) [11]	Closed	4 Hz, 17 Hz	Not mentioned	LED glasses and headphones	Not specified	Not specified	20 min (each stimulation interval)	Repeated exposure	Not specified
Timmermann et al. (1999) [12]	closed	Dominant alpha and twice dominant alpha	185 Hz sine wave	Polysync Pro Synetic Systems (headphones and photic glasses)	Not specified	Not specified	20 min per condition	2 sessions, each separated by 2 weeks	Red light (LEDs)
Roberts et al. (2018) [50]	Not specified	5.5 Hz	Not mentioned	Audio-visual entrainment (headphones and goggles)	Not specified	Not specified	36 min (entrainment session)	2 experiments with different entrainment conditions	Not specified
Cantor & Stevens (2009) [65]	Not specified	14 Hz	Not mentioned	Mind Gear PR-2x auditory-visual stimulation system (LED glasses and binaural beats)	Not specified	Not specified	30 min daily for 4 weeks	8 weeks total, including 4 weeks crossover	Green LEDs
Pino et al. (2022) [66]	Not specified	Not specified	Not specified	Neuro-Upper audio-visual entrainment system	Not specified	Not specified	30 min per session	55 sessions	Not specified
Berg & Siever (2009) [67]	Seasonal Affective Disorder (SAD)	1 Hz, 20 Hz	Not mentioned	DAVID Paradise AVE device (light and tone pulsing)	Not specified	60–70 dB (adjustable)	20 min (each session)	4 weeks (2 weeks placebo, 2 weeks active)	Not specified
Klimesch (1999) [68]	Not specified	Not specified	Not specified	EEG oscillations analysis (alpha and theta)	Not specified	Not specified	5 min per block	8 sessions, post-test session	Not specified
Hanslmayr et al. (2005) [69]	Not specified	Not specified	Not specified	Neurofeedback for upper alpha enhancement	Not specified	Not specified	5 min per session	4 weeks	Not specified
Pino & Romano (2022) [66]	Opened	Not specified	Not specified	Neuro-Upper audio-visual entrainment system	Not specified	Not specified	30 min per session	55 sessions	Not specified
Pearson & Wilbiks (2021) [70]	Not specified	2 Hz to 32 Hz	Not specified	Self-generated audiovisual memory cues	Not specified	Not specified	Not specified	6 months	Not specified
Seger et al. (2023) [71]	Not specified	5.5 Hz	Not mentioned	Virtual navigation + mental simulation	Not specified	Not specified	28–33 sessions, 22-min sessions	7 weeks	Not specified
Roberts et al. (2018) [50]	Not specified	7–9 Hz and 12–22 Hz	Not mentioned	DAVID PAL 36 by Mind Alive Inc. (headphones and visual stimulation)	Independent left and right visual stimulation	Not specified	20–30 min sessions	4 weeks	Not specified
Joyce & Siever (2000) [72]	Closed	10 Hz, 18 Hz	Not mentioned	DAVID Paradise XL (field independent eyeglasses)	Left-right field independent	Not specified	3-min sessions per condition	3 months	White light with light blue tint
Siever (2003) [73]	Closed	40 Hz	Binaural beats	DAVID Paradise XL, Light-sound stimulation device	Not specified	Not specified	30 min nightly for 4 weeks	1-week baseline, 4-week intervention	White light with light blue tint
Halpin et al. (2023) [74]	Opened	Delta, 2 Hz	Not specified	Smartphone app (hBET) using light and sound stimulation	Left-right visual stimulation	Not specified	30 minutes per session	6 months	Not specified
Chan et al. (2022) [75]	Opened	Not specified	Binaural beats	Procyon AVS device (light goggles and headphones)	Not specified	92% adherence, under user control	30 min nightly for 1 month	2 months	White flicker
Tang (2021) [76]	Closed	Not specified	Not specified	Polysync Pro Synetic Systems (headphones and photic glasses)	Not specified	Not specified	20 min per condition	1 month	White flicker
Tang (2015) [77]	Closed	Not specified	Not specified	Not specified	Not specified	Not specified	Not specified	2 weeks	White flicker

## 3. Safety

There is a notable scarcity of comprehensive research examining the safety of audiovisual entrainment. To date, specific adverse effects have not been documented. Nevertheless, two conditions warrant consideration. Photosensitive epilepsy emerges as a primary concern within this context. Epidemiological data suggest that approximately 5% of individuals with epilepsy are susceptible to photic-induced seizures, with higher incidence rates observed among younger demographics and women [78]. Consequently, this subgroup should be categorically excluded from AVE interventions to prevent the risk of seizures. In one of the studies in phase one the safety and feasibility of 40 Hz has been examined, including 2 patients with epilepsy. During a brief session no abnormal brain activity or discomfort has been reported by 40 Hz stimulation [31]. However, further investigation is necessary regarding epilepsy, as another recent study has not only refrained from excluding it but has also successfully introduced audiovisual entrainment as a potential treatment for the condition [30].

Another condition that warrants consideration is migraine, particularly photic- or visually triggered migraine. Repetitive light stimulation has been reported as a potential trigger for migraine attacks in susceptible individuals [79,80]. While AVE protocols have not systematically documented migraine induction, the possibility of exacerbating symptoms should be acknowledged, and individuals with a history of visually induced migraine should be carefully monitored or excluded from such interventions.

Moreover, the implications of excessive AVE use have been studied in animal models, revealing significant neurobiological consequences. Research conducted by Mansouri [7] indicates that overexposure to AVE in rats increases neuronal density in the amygdala, which correlates with a marked decrease in social behavior. These findings underscore the need for regulated use and highlight AVE’s potential to alter brain function adversely.

Considering these facts, it is imperative that future research rigorously explores the safety profile of AVE. This will not only delineate the boundaries of safe use but also ensure that AVE’s therapeutic applications are both practical and safe for all user groups, especially those at risk of adverse effects.

## 4. Area of Application

### 4.1. Depression

Depression is a global mental disorder with a prevalence of an estimated 5% of adults [81]. This condition extensively disrupts daily functioning, manifesting through various symptoms, including cognitive impairment, attention deficits, verbal and nonverbal learning, short-term and working memory, auditory and visual processing, problem-solving, processing speed, and motor skills [82]. According to a conducted study, the societal cost of depressive disorders in Europe was estimated at approximately EUR 6145 million [83], with an average price per patient per year of EUR 3402 [83,84]. Based on 65 solid studies from 79 countries, the average treatment rate for this disease is 38.8% within 12 months, with significant differences between high-income countries (31.5%) and low-income countries [85].

To evaluate the efficacy of audiovisual entrainment (AVE), 16 participants underwent 20 sessions, each lasting 30 min per day (5 days per week) for four weeks. In treating depression, participants were subjected to 14 Hz stimulation, while the control group received relaxation music without any visual stimulation for five weeks. Significant outcomes include a reduction in depressive symptoms, positioning AVE as a promising non-pharmacological intervention [65].

In another study with a closed-loop design, 15 participants underwent 52 sessions, each session 30 min. Based on the real-time EEG (real-time feedback) from the participants, the audiovisual stimuli presented in alpha (7.5–11.75 Hz) to induce relaxation and Beta (12–31 Hz) for cognitive enhancement and mood regulation. Significant reduction in depressive symptoms, with participants showing improvements in Hamilton Rating Scale for Depression (HAM-D) scores after the intervention was reported [86]. Another study on depression indicated notable changes in depression measured by HAM-D. The study recruited 15 participants with anxiety and depressive symptoms and divided them into 8 for the experiment and 7 in the control group. Within 52 sessions (5 sessions per week), for 45 min online EEG feedback base [87].

### 4.2. Seasonal Affective Disorder

Seasonal affective disorder (SAD) is a type of depression that occurs after shifting to the primary winter months [88]. The prevalence is 6%, and according to the geographical location, the prevalence varies. This disorder is more seen in winter months [89].

An investigation divided 74 affected participants into 16 in the control group and 58 who received two weeks of 20 Hz entrainment. The AVE group showed significantly reduced depression and anxiety symptoms. It also improved social interactions within family and work environments, enhancing happiness and energy levels. Moreover, there was a significant decrease in eating and appetite [67].

### 4.3. Insomnia

Insomnia, affecting up to 10% of the adult population in Europe, presents significant public health concerns [90]. Studies have demonstrated notable differences in the high-frequency EEG spectral power density of sleep among those with and without insomnia, suggesting neurophysiological disparities as potential underlying causes [91,92]. A recent study analyzing the EEG power spectrum of 1985 participants reproved this statement again [93]. Different symptoms have been reported based on the period of the insomnia. Furthermore, all-cause mortality rates are heightened in men with chronic sleep disturbances [94].

Regarding therapeutic interventions, the use of an Audiovisual Stimulation (AVS) device over four weeks has been shown to yield significant improvements in insomnia symptoms and associated pain. The device operates through 30 min sessions of light flickering (goggles) and sound pulsing (headphones), which gradually transition from alpha (8 Hz) to delta (1 Hz) frequencies, effectively entraining brainwaves towards a state of deep relaxation and sleep [9]. A specific application of this technology in older adults over one month resulted in a significant decrease in insomnia severity, shifting from clinically moderate to sub-threshold (mild) insomnia [77]. These findings highlight the potential of AVS as a non-pharmacological treatment option for insomnia, offering substantial benefits across different age groups.

### 4.4. Cognitive Functions

Thinking, reasoning, language, memory, problem-solving, decision-making, and attention constitute core aspects of human cognitive domains [95]. Impairments in these functions are called cognitive deficits, which can stem from various causes. While such deficits are not classified as a disorder, they may indicate an underlying condition.

Throughout various tasks or cognitive states, the brain modifies the ratio of the frequency bands. For example, during cognitive tasks such as problem-solving and concentration, the predominant wave observed is beta waves (13–30 Hz) [96]. Alpha waves (8–12 Hz) are prevalent during relaxed states and light meditation [68]. The theta band (4–8 Hz) predominates during more profound relaxation or light sleep and is associated with creativity, intuition, and daydreaming [69]. However, the brain state and the activated regions are more complex. These studies provided insights for the creation of AVE procedures.

Several studies have explored the effectiveness of AVE in addressing these cognitive impairments. Aiming to improve cognitive function, the research included 15 participants who completed 52 consecutive sessions, each lasting 30 min, conducted daily from Monday to Friday. The research presented AVE in frequencies: Delta (0.5–2.75 Hz), Theta (3.5–6.75 Hz), Alpha 1 (7.5–9.25 Hz), Alpha 2 (10–11.75 Hz), Beta 1 (13–16.75 Hz), Beta 2 (18–29.75 Hz), Gamma 1 (31–39.75 Hz), and Gamma 2 (41–49.75 Hz). A control group of 8 participants was assigned to a waitlist and engaged solely in self-help groups. Results indicated a considerable enhancement in cognitive function measured by the Performance Intelligence Quotient (QIP) [86]. Another study with an RCT design and 15 participants, 7 for the treatment and 8 for the control group of depressed participants, measured the cognitive function by Cognitive functions by Wechsler Adult Intelligence Scale-Revised (WAIS-R) and Raven’s Progressive Matrices (RPM). After 52 sessions of giving AVE based on EEG online feedback in the frequency range of 0.5–49.75 Hz, the participants experienced significant improvements in cognitive functions. AVE is supposed to be a neurofeedback device compiled with EEG to monitor the brain state. A study examined the impact of audiovisual entrainment as neurofeedback on cognitive functioning in 18 psychiatric disorders. Participants completed 55 neurofeedback sessions and audiovisual entrainment treatments, recording brainwave activity. Results showed significant improvement in IQ scores for 16 out of 18 participants [66].

#### 4.4.1. Memory

Memory is a pivotal mental process that encompasses the encoding, storage, and retrieval of information and plays a crucial role in learning and personal identity formation.

Research employing AVE to enhance memory functions has yielded promising results. Notably, theta band power (4–8 Hz) increases during memory-related activities [70,71,97,98]. It has been theorized that inducing theta band activity could improve memory abilities. Empirical studies with AVE have confirmed that theta induction (5.5 Hz) significantly improves memory, whereas inducing beta waves (14 Hz) does not yield significant differences [49,50]. Additionally, stimulation in 3 min of 40 Hz has been examined, and equal to the beta stimulation, no significant change has been reported [6].

#### 4.4.2. Attention

Attention constitutes a critical aspect of cognitive functioning, encompassing the deliberate allocation of mental resources to specific objects, concepts, tasks, and particular elements within the environment. This cognitive process is essential for human consciousness and perception, facilitating information processing, decision-making, and communication while optimizing finite mental resources. Attention Deficit Hyperactivity Disorder (ADHD) represents a prevalent neuropsychiatric condition, with an incidence of 7.5% among children and adolescents (10% in boys and 5% in girls) [99] and 3.10% in adults [100]. The annual economic burden of ADHD in Europe is approximately 12,171€. This condition is associated with various comorbidities and adverse outcomes [101]. Electroencephalogram (EEG) biomarkers for ADHD have revealed notably higher average delta (0–4 Hz) and theta (4–8 Hz) bands and lower beta (13–20 Hz) (Theta/Beta ratio) in the frontal regions [102,103].

In an early investigation, thirty-four elementary school students had AVE treatment for seven weeks, each lasting around twenty minutes. The subjects underwent two distinct protocols. The initial eight sessions at low-alpha (7–9 Hz) given across 20 min are designed to facilitate relaxation. The remaining sessions for SMR (sensory motor rhythm) (12–15 Hz) and beta (15–19 Hz) will last 22 min each, and significant improvements in inattention and impulsivity will be reported [72]. In a subsequent study, school students participated in 30 sessions of AVE, lasting 20–30 min each, two or three times per week. The equal protocols employed initially ranged from 7 to 9 Hz to induce relaxation, followed by 13–18 Hz to increase brain arousal, resulting in marked improvements [104].

Unilateral spatial neglect is a disease that leads to significant complications. It involves the brain’s failure to acknowledge or respond to stimuli on one (usually the left) side of the environment [105]. AVE has been used as a therapeutic approach for this condition. Following ten daily training sessions, totaling four hours each over two weeks, results demonstrated improved visual exploration and a reduction in symptoms of neglect [31].

#### 4.4.3. Alzheimer’s Disease

Alzheimer’s disease (AD) is a degenerative condition that compromises behavioral function and interferes with daily activities [106]. Among the 0.7% of adults who have dementia, 60 to 80% progress to Alzheimer’s disease, while the disorder is becoming prevalent worldwide [107]. This disorder not only hinders the individual from leading an everyday life but also manifests symptoms such as agitation, anxiety, and melancholy, imposing significant emotional and financial burdens on families due to caregiving, which leads to stress, burnout, and inadequate support [108]. The yearly personal expense of dementia was substantial, averaging $23,796 per individual in 2019. The increased cost places considerable pressure on families, especially in low- and middle-income countries, where a large share of care is unpaid and informal [109]. Amyloid beta has been identified as a primary marker of Alzheimer’s disease in these illnesses [110]. The detected biomarkers of Alzheimer’s in EEG findings included increasing amplitude of Delta and Theta (1–8 Hz) band and decreasing in Alpha (8–12 Hz) and Beta (8–30 Hz), mainly in occipital and parietooccipital areas [111].

The studies in the mice model have shown promising results, and in the first studies, stimulation involved only 40 Hz light flicker (Without Auditory stimulation), causing a reduction in tau hyperphosphorylation, a significant decrease in Amyloid-β plaques in the hippocampus, and improvements in the circadian rhythm disturbances in AD mice [112,113,114]. A randomized, placebo-controlled pilot trial recruited 15 persons with moderate probable Alzheimer’s dementia to determine if similar effects would be observed in individuals. The results were inconclusive after three months of daily one-hour 40 Hz multi-sensory stimulation sessions. The initial effect of 40 Hz stimulation was shown in atrophy. Compared to the control group exposed to continual light and white noise, no substantial ventricular dilatation or hippocampus atrophy was observed, whereas the control group exhibited notable changes. The second finding indicated increased functional connectivity within the default mode network (DMN), whereas the control group demonstrated decreased connectivity. There has been a considerable improvement in cognitive function on the face-name association delayed recall test comparing the two groups. The final effect of AVE was on the regularity of disruptions in everyday activity patterns, which serves as a marker for Alzheimer’s disease. Substantial enhancement, as indicated by the Inter-daily Stability (IS) indicator, was observed during three months [75].

##### Pain

Chronic pain, a persistent and severe health issue, originates from various sources and affects approximately 27.5% of the global population [115]. This condition significantly impacts multiple aspects of daily life. AVE has been employed to alleviate pain and its associated effects. Initial research in this field indicated significant reductions in medication use, suicidal thoughts, and stress, alongside increases in hope, self-esteem, and improvements in family dynamics [116].

The pilot research involved nine participants independently engaged in a 30 min AVS program each night at bedtime for one month. The results indicated that AVS may serve as a valuable intervention for enhancing and alleviating pain symptoms in persons with chronic pain [9]. A more recent investigation recruited 28 participants. The result demonstrated a significant reduction in pain after administering 10 Hz AVE before sleep for 30 min over four weeks [74].

Fibromyalgia is a disorder with a prevalence of 3.3% in the population. It is a multivariate in the symptoms. The leading indicators are musculoskeletal pain, fatigue, cognitive difficulties, and sleep disorders; their severity increases with aging. This disorder is more prevalent among women than men [117]. A study evaluated the efficacy of three interventions for Fibromyalgia Syndrome. Forty-nine people with this disorder were randomized to the AVE, medicine, and nutrition groups. AVE was administered daily, three times for 30 min each, in the morning with Beta (12–30 Hz), in the afternoon with Alpha (8–12 Hz), and in the evening with Delta (0.5–4 Hz) and Theta (4–8 Hz) frequencies for one month. The AVE group exhibited substantial enhancement in all assessed variables (anxiety, pain, fatigue), but the medication group demonstrated more significant improvement [118].

## 5. Mechanism of Action

Based on these clinical findings, it would be valuable to further investigate the underlying mechanisms of the effects of AVE. A deeper understanding could be achieved by clarifying the pathways involved in both the auditory and visual systems. Unlike other techniques AVE comprehensively covers most of the brain regions and among the neuromodulatory techniques seems to engage most regions of the brain. To grasp the effects of AVE, it is essential to understand the sensory pathways it engages in. Visual stimuli travel from the retina through the optic nerve to the lateral geniculate nucleus (LGN) and the primary visual cortex (V1), where initial processing occurs. Further analysis happens in specialized areas such as V2, V3, and the middle temporal region, which handle complex tasks like motion and object recognition. Similarly, auditory stimuli enter the cochlea, converting sound waves into electrical signals transmitted via the auditory nerve to the auditory cortex [119]. The reason for AVE’s widespread effects is its ability to engage most brain regions and synchronize them naturally with the stimuli. This unique ability highlights AVE as an excellent and natural form of brain modulation that sets it apart from other techniques like NiBS, which are electrical-based, less comprehensive, and less intrinsic in their approach.

### EEG

The seminal discovery of audiovisual entrainment (AVE) occurred after electroencephalography (EEG) became available in the late 1920s. Initial research, notably by Adrian and Mattews [64], demonstrated that brain rhythms could be modified through exposure to flickering lights at various frequencies. This foundational study is credited with pioneering the investigation of flickering light’s effects on neural activity, catalyzing further research. Subsequent studies have rigorously examined the efficacy of this technique across different disorders.

The electroencephalogram (EEG) is a technique to monitor brain activity that stands out due to its cost-effectiveness and ability to record signals with high temporal resolution. This attribute makes it particularly useful for observing the effects of audiovisual entrainment (AVE). A pioneering study employing EEG in this domain utilized three protocols at 1 Hz and 12 Hz frequencies and a dynamic decrement from 12 to 1 Hz over 7.5 min [5]. This resulted in the documentation of two extracted features. The alpha band power (8–12 Hz) considerably decreased across every intervention, while variations in the other bands were seen but not deemed significant. Dipole sources were computed, revealing variations across different bands. In the theta band (4–7.5 Hz) across all procedures, the dipole source shifted posteriorly and superiorly during stimulation. A leftward shift during protocol stimulation and a rightward and superior shift have been recorded, with more activity in the right hemisphere during beta band (12.5–20.5 Hz) stimulation [5]. Variability in EEG responses to AVE has been noted, especially among individuals with different baseline alpha powers. This was observed after presenting 7.5 min of AVE in low, high, and combined intensities and variations. Alpha stimulation (10 Hz) and beta stimulation (22 Hz) enhance alpha power at both Cz and Pz but with varying effects in participants with high baseline alpha power. High baseline alpha power was associated with lower alpha entrainment and varied responses to stimulation. Beta stimulation’s effects were influenced by both baseline alpha and beta power levels, with higher baseline beta and alpha power contributing to the observed effects. The presentation of stimuli at this frequency enhanced the power of other oscillatory bands (delta 1, 0.75–2 Hz; delta 2, 2–4 Hz; theta, 4–8 Hz; alpha, 8–12 Hz; beta 1, 12–21 Hz; and beta 2, 21–31 Hz), with effects persisting in the Beta 1 band up to 30 min post-stimulation [12].

EEG synchrony under audiovisual stimulation (AVS) by 4 and 20 Hz was investigated using Wavelet transform-derived instantaneous phases. Phase synchronization, measured by phase difference uniformity, increased significantly during AVS compared to non-stimulation across all cortex locations. The minor increases were in the frontal areas, while the central region maintained high synchronization levels similar to visual processing centers in the posterior cortex [11].

In another study, fourteen volunteers formed a study group that underwent 16 sessions of combined auditory-visual stimulation delivered via mobile phone over four weeks. The primary objective of these sessions was to enhance alpha peak activity. However, upon conducting a power analysis, the results did not indicate any significant changes in alpha peak activity [8]. This outcome suggests that the intended modulatory effects of auditory-visual stimulation on alpha-peak activity may not be as readily achievable under the conditions tested in this study. Fourteen volunteers received 16 sessions of combined auditory-visual stimulation in patterns of alpha and beta bands (7.8, 8.8, 12.8, 13.4, 14.4, 18, 19, and 23 Hz) over four weeks. In comparison, seven volunteers in the control group received auditory-only stimulation for two sessions. EEG recordings were taken at the beginning and end of the study to measure individual alpha peak frequencies (iAPF) and assess entrainment and resonance effects. Resting-state recordings (eyes open and eyes closed) during two lab sessions, one in week one and one in week four, were conducted for five minutes each before and after the stimulation session. Each stimulation session lasted approximately 16 min and consisted of three repetitions of the visual and auditory stimulation patterns. The study found no significant differences in individual alpha peak frequencies (iAPF) between week one and week four for the study and control groups. These first EEG studies suggested that AVE may interact with brain oscillatory behavior, which may be a proxy for treating diseases showing altered brain rhythms [8].

## 6. The Current State of AVE

Although AVE is not a new technique, it is still young scientifically, as it remains insufficiently standardized and has not yet been systematically studied. Current findings suggest predominantly positive outcomes, highlighting AVE’s promise as a therapeutic intervention that warrants further detailed investigation. At the same time, the available evidence is not uniformly positive. For example, it has been found that AVS substantially improved mood and mood-sensitive cognitive tasks, but the effects were largely restricted to self-reported affective states rather than broader clinical outcomes [120]. A non-controlled feasibility study reported within-participant improvements in sleep, pain, fatigue, and mood following pre-sleep alpha entrainment, yet the absence of a control group limited conclusions about specific AVE effects [74]. In contrast, a pilot randomized trial showed that although older adults with comorbid insomnia and osteoarthritis pain reported improvements in sleep and mood, these changes were also present in the placebo group, resulting in no significant between-group differences [76]. Finally, another study demonstrated across multiple experiments that 40 Hz audiovisual stimulation did not enhance visual thresholds or spatial memory, with observed changes primarily reflecting practice effects [6]. Taken together, these findings illustrate that while AVE holds promise, its effects are not universal and may vary depending on study design, outcome measures, and target population.

This review represents one of the first attempts to provide a structured framework for AVE by disentangling its components and modes of stimulation. Such a framework may enable future studies and reviews to evaluate AVE with greater precision, as most prior investigations have failed to report these parameters comprehensively. However, many of the materials and concepts applied here are indirect, borrowed from other neuromodulation techniques. Future work should directly examine the essential parameters of AVE including the main stimulation color, primary frequency bands (delta, theta, alpha, beta sub-bands, and gamma), phase offsets, signal shape, and differences between right and left visual field stimulation rather than relying on extrapolations.

Beyond these structural considerations, several empirical uncertainties remain unresolved. For example, it is still unknown how long stimulation must be applied within a single session to achieve maximal effects, how long such effects persist after stimulation, whether repeated sessions lead to habituation or sensitization, and what the long-term consequences may be. One study suggested that even a short exposure of five minutes may be sufficient, although this finding was limited to improving mood rather than broader clinical outcomes [116], making it insufficient to establish generalizable conclusions. Another study reported that no further changes occurred after the first week of intervention [7], yet this cannot be taken as evidence that one week represents the optimal duration of AVE. Rather, these findings highlight the current lack of consensus and underscore that the effective dosing and scheduling of AVE remain largely unknown parameters that require systematic investigation.

The safety profile of AVE also requires further clarification. Although existing studies suggest that the technique is generally well tolerated, the number of systematic safety evaluations is limited. More rigorous monitoring of adverse events and reports of non-effectiveness would help delineate the boundaries of safe and effective application.

AVE can be viewed as a practical application of DAT, as it uses timed auditory and visual pulses to guide neural and attentional rhythms. From a DAT perspective, such stimulation provides the temporal coherence needed for attunement, strengthening temporal expectancies and supporting future-oriented attending. This makes AVE a promising tool for scaffolding attentional mechanisms involved in language, reading, and other timing dependent skills. However, studies that have relied on DAT have mostly used auditory pulses [121,122,123,124,125,126,127], and no structured audiovisual rhythmic approach as introduced in this paper has yet been applied to test this theory directly. A detailed study examining the link between DAT and AVE would therefore be highly valuable and could substantially strengthen the theoretical and practical foundations of this technique.

Another challenge lies in the incomplete mechanistic understanding of AVE. Phenomena such as the “fusion illusion” where mismatched auditory and visual stimuli alter perceptual outcomes highlight the complexity of audiovisual integration [117,119]. Moreover, the possibility of combining AVE with other sensory modalities, such as rhythmic tactile stimulation, remains largely unexplored and could open new research avenues.

In summary, this review has sought to establish a clearer structure for AVE and to encourage systematic scientific investigation. While existing studies highlight substantial potential for benefits across mood, sleep, pain, and cognition, the evidence also remains mixed, with several investigations reporting null or limited effects depending on outcome measures and study design. This underscores the need for more rigorous, standardized, and controlled research before AVE can be regarded as a reliable therapeutic approach. Providing a framework for its parameters and modes of application is therefore an important step toward greater consistency in future trials and, ultimately, the establishment of its clinical value.

## Figures and Tables

**Table 1:** AVE parameter reporting checklist and selection guide. This table outlines the key parameters currently understood to influence Audiovisual Entrainment (AVE). Including and clearly reporting these conditions in future research may support greater methodological consistency and contribute to the gradual development of standardized AVE protocols.

	Visual Condition	Monocular/ Binocular	Visual Field	Frequency	Phase	Presentation Mode	Color
Visual Stimuli	EC	Binocu lar Visual Exposure	RVF	X	0–1	Static Frequency	X
	EO	Unilateral Visual Exposure	LVF	VLF = X RVF = Y		Ramp Up/Down	X for RVF Y for LVF
			RVF/LVF			Random Frequency Range	X for Left Eye Y for Right Eye
						Predefined Frequency Range	
	**Experimental Condition**	**Binaural**	**Binaural/** **Monaural Beats**	**Frequency**	**Presentation Mode**		
Auditory Stimuli	Binaural	Spontaneous Bilateral	Auditory Pitch Difference	X	Static Frequency		
		or Altering Unilateral		X for Left Y for Right	Ramp Up/Down		
					Random Frequency Range		
					Predefined Frequency Range		
	Monaural						

## Abbreviations

The following abbreviations are used in this manuscript:

AVE	Audiovisual Entrainment
AVS	Audiovisual Stimulation
AD	Alzheimer’s Disease
BWE	Brainwave Entrainment
DAT	Dynamic Attending Theory
LSM	Light and Sound Machines
NiBS	Non-invasive Brain Stimulation
tDCS	Transcranial Direct Current Stimulation
rTMS	Repetitive Transcranial Magnetic Stimulation
LGN	Lateral Geniculate Nucleus
RVF	Right Visual Field
LVF	Left Visual Field
BB	Binaural Beats
MB	Monaural Beats
tACS	Transcranial Alternating Current Stimulation
tRNS	Transcranial Random Noise Stimulation
EC	Eyes Closed
EO	Eyes Open
EEG	Electroencephalography
iAPF	Individual Alpha Peak Frequency
HAMD	Hamilton Rating Scale for Depression
SAD	Seasonal Affective Disorder
QIP	Performance Intelligence Quotient
WAISR	Wechsler Adult Intelligence Scale Revised
RPM	Raven’s Progressive Matrices
SMR	Sensory Motor Rhythm
